# Using machine learning algorithms to review computed tomography scans and assess risk for cardiovascular disease: Retrospective analysis from the National Lung Screening Trial (NLST)

**DOI:** 10.1371/journal.pone.0236021

**Published:** 2020-08-03

**Authors:** Amos Stemmer, Ran Shadmi, Orna Bregman-Amitai, David Chettrit, Denitza Blagev, Mila Orlovsky, Lisa Deutsch, Eldad Elnekave

**Affiliations:** 1 Sackler School of Medicine, Tel Aviv University, Tel Aviv, Israel; 2 Zebra Medical Vision, Ltd, Shfayim, Israel; 3 Pulmonary and Critical Care Division, Intermountain Medical Center, Murray, Utah, United States of America; 4 Pulmonary and Critical Care Division, University of Utah, Salt Lake City, Utah, United States of America; 5 BioStats Statistical Consulting Ltd, Maccabim, Merkaz Renanim, Israel; 6 Department of Diagnostic Radiology, Rabin Medical Center, Beilinson Hospital, Petah Tikva, Israel; Universite de Bretagne Occidentale, FRANCE

## Abstract

**Background:**

The National Lung Screening Trial (NLST) demonstrated that annual screening with low dose CT in high-risk population was associated with reduction in lung cancer mortality. Nonetheless, the leading cause of mortality in the study was from cardiovascular diseases.

**Purpose:**

To determine whether the used machine learning automatic algorithms assessing coronary calcium score (CCS), level of liver steatosis and emphysema percentage in the lungs are good predictors of cardiovascular disease (CVD) mortality and incidence when applied on low dose CT scans.

**Materials and methods:**

Three fully automated machine learning algorithms were used to assess CCS, level of liver steatosis and emphysema percentage in the lung. The algorithms were used on low-dose computed tomography scans acquired from 12,332 participants in NLST.

**Results:**

In a multivariate analysis, association between the three algorithm scores and CVD mortality have shown an OR of 1.72 (p = 0.003), 2.62 (p < 0.0001) for CCS scores of 101–400 and above 400 respectively, and an OR of 1.12 (p = 0.044) for level of liver steatosis. Similar results were shown for the incidence of CVD, OR of 1.96 (p < 0.0001), 4.94 (p < 0.0001) for CCS scores of 101–400 and above 400 respectively. Also, emphysema percentage demonstrated an OR of 0.89 (p < 0.0001). Similar results are shown for univariate analyses of the algorithms.

**Conclusion:**

The three automated machine learning algorithms could help physicians to assess the incidence and risk of CVD mortality in this specific population. Application of these algorithms to existing LDCT scans can provide valuable health care information and assist in future research.

## Introduction

The National Lung Screening Trial (NLST) was conducted to determine whether annual screening with low-dose computed tomography (LDCT) scans could reduce mortality from lung cancer in high-risk individuals [1]. In the NLST, more people died of cardiovascular disease (24.8%) than lung cancer (24.1%) (1). This result is consistent with the repeated observations that cardiovascular disease (CVD) is the leading cause of death in the 21^st^ century [2] and the leading cause of death of smokers [3].

Routine measurement of the Coronary Calcium Score (CCS), a well-established risk factor for CVD quantified by the Agatston score [4–6], has been shown to detect CVD in LDCT scans. This type of CVD screening has been shown to lead to finding 84 patients with a CCS above 1000 out of 1000 patients screened, who could benefit from starting secondary preventive medical treatment of CVD such as antihypertensive or lipid lowering drugs or both, that they didn’t receive before [7]. In addition, LDCT could also identify fatty liver, which is independently associated with increased CVD risk [8–10]. Finally, a specific diagnosis of emphysema can also be made from the screening LDCT, which is by itself a risk factor for CVD [11], this screening could also lead to other disease specific interventions for pulmonary disease as well as CVD.

Although early diagnostics based on CT screening is very effective, the current approach, which relies primarily on manual evaluation of medical imaging is limited due to varied interobserver reliability [12–14] and limited time for scan [15]. With recent advances in artificial intelligence (AI) and machine learning algorithms, using automation to immediately and accurately analyze each CT scan and create query-able, consistent interpretations is closer than ever. Such algorithms, if implemented appropriately, could assist physicians and lead to early diagnosis and improved patient care.

In this study, we aimed to apply three automated machine learning algorithms that provide CCS, liver steatosis and emphysema percentage in the lungs to low-dose CT scans, in order to assess the utility of these tools as predictors of CVD incidence and mortality.

## Materials and methods

### Data acquisition

The study was submitted to review by the NCI (National Cancer institute) and was approved as NLST-246.

**Fig 1:**
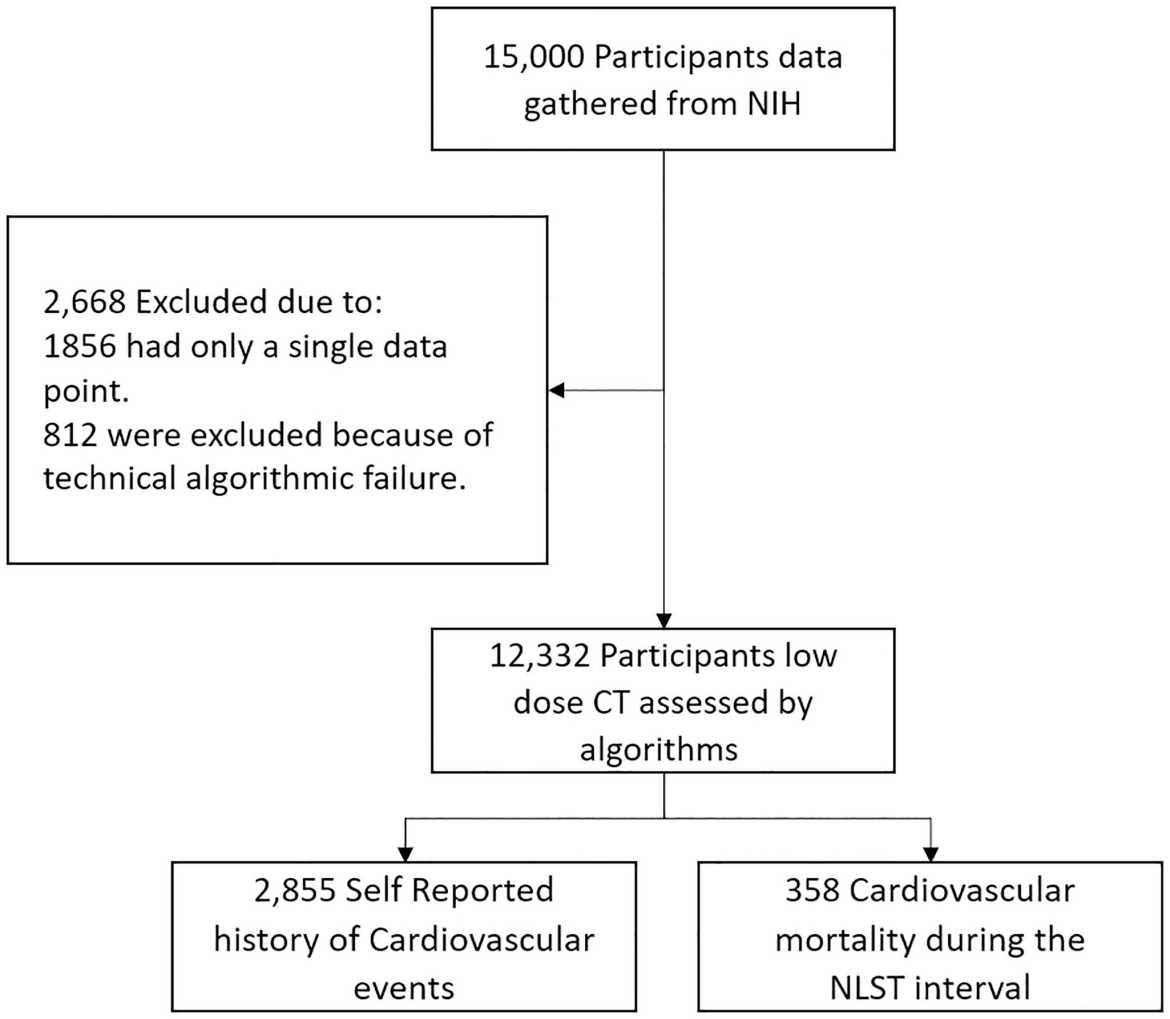
Data flow chart.

Data from 15,000 participants of the NLST trial were acquired. For each participant three LDCT screenings (T0, T1, T2) were requested, each screening was made at 1-year intervals. These participants were 55–74 years old with a history of cigarette smoking of at least 30 pack years, and, if former smokers, had quit within the previous 15 years [1]. Participants who met the NLST exclusion criteria [1], were screened with an annual Chest X-ray rather than LDCT or did not complete the NLST study, were not selected. A sample of 15,000 participants was received from the NIH, representing a dataset enriched for subjects who had self-reported cardiovascular events and those who died of cardiovascular disease during the NLST interval. Subject data was excluded (Fig 1) if it contained only a single LDCT time point or in the event that algorithmic assessment of either Coronary Calcium, Liver Density or Quantitative Emphysema was technically unsuccessful. Median duration of follow up was 6.5 years with a maximum duration of 7.4 years.

### Algorithms used

In this study we used three automated machine learning algorithms which were designed to assess biomarkers on non-contrast chest CT, specifically the percent emphysema (Emphy-Alg), liver density (LD-Alg) and quantity of coronary calcium (CCS-Alg).

CCS-Alg is an algorithm designed to assist radiologists in evaluating coronary artery calcification (CAC) by providing CCS and its quantification the Agatston score from CT scans as was previously published [16]. CCS is measured as a continuous score while Agatston is reported in categories.

The Emphy-Alg is an algorithm for analyzing images from CT thoracic scans. This application performs automatic segmentation and volume calculation of the pulmonary tissue from non-contrast thoracic CT scans and quantifies the volume of low-attenuation air in the lungs (low-attenuation volume is defined as volume with attenuation lower than -950 Hounsfield units (HU) [17]. The application provides the low-attenuation volume as a percentage of the overall lung volume, this allows us to assess the percentage of emphysema.

LD-Alg is an algorithm which automatically analyzes pre-existing chest and abdomen CT scans (with or without a contrast agent) that include part of the liver and assesses liver attenuation. This algorithm accepts CT scans (DICOM files) and provides an averaged HU value of the liver. Steatosis will cause a reduced HU value [18] and LD-Alg score. The LD-Alg score is provided as a continuous score.

### Statistical methods

Statistical analyses were performed in SAS v9.4 (SAS institute, Cary, NC, USA) and in R v3.5.1.

The first valid measurement of each of the scores (CCS-Alg, Emphy-Alg, and LD-Alg) was used in the analysis. Logistic regression was used to determine whether each of the 3 scores is associated with the outcomes of interest. The 3 scores were modeled individually and in multivariate logistic regression models together with age, gender and pack years. The CCS-Alg score was modeled both as a 5-level categorical variable similar to the Agatston score scale and as a continuous score. The Emphy-Alg and LD-Alg scores were modeled as continuous scores with odds ratios (OR) reported for 10% or 10-unit increments of each respectively. The area under the ROC curve (AUC) was used as a measure of the discriminatory power of the models. Nested models were compared by the difference between AUC as well as the correlated 95% confidence interval [19]. Model parameters with level of significance are presented. ORs and 95% confidence intervals (CI) for both univariate and multivariate models are also presented. A *P* value < 0.05 was considered statistically significant, nominal p-values are presented.

## Results

### Demographics

The final analysis included 12,332 participants (Fig 1). The majority of participants were male (*n* = 7308, 59.3%) mean (SD) age was 61.9 (5.1), females (*n* = 5024, 40.7%) mean (SD) age was 61.5 (5), and overall, the mean (SD) age was 61.8 (5.1) years (range, 55–74 years) (Table 1). All patients have a history of cigarette smoking (of at least 30 pack years or had stopped within 15 years before the initiation of the NLST study), with mean (SD) of cigarette packs per year of 56.3 (24.1) and range of 30–295 cigarette pack years (Table 1).

**Table 1:** Characteristics of the study population.

	Survived *n* = 11322	CVD incidences *n* = 2855	CVD mortality *n* = 358	*All participants n* = 12332
Age	61.6(5)	62.9 (5.2)	63.8 (5.52)	61.8 (5.1)
Pack years	55.6 (23.7)	60.8 (26.7)	63.7 (26.7)	56.3 (24.1)
Females n (%)	42	29.1	32	
CCS-Alg	291.4 (569.77)	664 (868.4)	607.11 (857.3)	313.2 (603.2)
Emphy-Alg	3.38 (7.31)	3.2 (6.8)	2.8 (6.6)	3.4 (7.4)
LD-Alg	44.6 (12.6)	42.6 (27.8)	29.5 (29.9)	43.4 (28)

Except where indicated, data are mean (SD).

The clinical outcomes of interest in this population were CVD incidence (*n* = 2855, 23.2%) and CVD mortality (*n* = 358, 2.9%) (Table 1). CCS-Alg Agatston categories stayed the same throughout the 3 time points in 52.7% (*n* = 6505) of the participants. Of the remaining, 21% (n = 2592) changed 1 group over and 26.2% (n = 3235) changed more than one group. Among those who had a change, the net movement from T0 to T2 was increased in 25.9% (n = 3196) and decreased in 21.3% (n = 2631). The biggest shift in categories was between Agatston category 3 in T1 to Agatston category 4 in T3, 5.8% (n = 724).

Data from 15,000 participants was gathered under NIH protocol NLST 246. 1856 (12%) participants were excluded because data only existed for a single time point and thus could not be analyzed for temporal dynamics. An additional 812 (5%) were excluded because of technical algorithmic failure pertaining to any one of the three automatic assessments of Coronary Calcium, Liver Density or Quantitative Emphysema.

### CVD mortality

#### Univariate analyses

A statistically significant association between CCS-Alg categories and CVD mortality was observed, with approximately 1.5-fold, 2-fold and 3.5-fold increased risk of CVD mortality in categories 11–100 (*n* = 2742), 101–400 (*n* = 2374), and >400 (*n* = 2780) vs 0 (*n* = 3451), respectively, and when treated as a continuous variable CCS-Alg demonstrated a 1.05-fold (P < 0.0001) increased risk for CVD mortality for every 100-unit increase in score (Table 2). A statistically significant association was also found between Emphy-Alg score and CVD mortality, with 1.14-fold (95% CI, 1.03–1.26) increased risk of CVD mortality for each 10% increment in the Emphy-Alg score (*P* = .0146 S1 Table in S1 Appendix), and between LD-Alg score and CVD mortality, with 1.16-fold (95% CI, 1.04–1.29) increased risk of CVD mortality for each 10 HU reduction in the LD-Alg score (*P* = .0077 S1 Table in S1 Appendix).

**Table 2:** Univariate analysis evaluating the association between CCS-Alg and CVD mortality and incidence.

	Level	OR estimate	95% confidence limits		*P*-value
CVD mortality	1–10 vs 0	0.96	0.54	1.70	.8750
	11–100 vs 0	1.48	1.03	2.12	.0361
	101-400vs 0	2.01	1.42	2.86	<.0001
	>400 vs 0	3.50	2.56	4.79	<.0001
	As continuous (100-unit increments)	1.05	1.03	1.06	<.0001
CVD Incidence	1–10 vs 0	0.82	0.65	1.02	.0722
	11–100 vs 0	1.11	0.96	1.29	.1460
	101–400 vs 0	2.18	1.90	2.50	<.0001
	>400 vs 0	5.91	5.22	6.68	<.0001
	As continuous (100-unit increments)	1.12	1.11	1.13	<.0001

CVD = cardiovascular disease, OR = odds ratio.

#### Multivariate models

The relationship between CVD mortality and CCS-Alg, Emphy-Alg, and LD-Alg was further explored via multivariate logistic regression together with age, gender and pack years which are known risk factors (Table 3). The AUC for this model was 0.6724 (95% CI, 0.64–0.7), suggesting low-to-moderate discrimination ability (Fig 1). The analysis demonstrated an increased CVD mortality risk of approximately 1.71-fold and 2.6-fold in CCS-Alg categories 101–400 and >400 vs 0, respectively. There was no increased risk in the 1–10 or the 11–100 category vs 0. The analysis also demonstrated approximately 1.12-fold increased risk of CVD mortality per 10 HU units decrease in LD-Alg scores (Table 3).

**Table 3:** Multivariate analysis evaluating the association between CCS-Alg categories, Emphy-Alg, LD-Alg, age, gender, pack years and CVD mortality.

Level	OR estimate	95% confidence limits		*P*-value
CCS-Alg 1–10 vs 0	0.99	0.53	1.72	.9845
CCS-Alg 11–100 vs 0	1.37	0.95	1.98	.0887
CCS-Alg 101–400 vs 0	1.72	1.2	2.46	.003
CCS-Alg >400 vs 0	2.62	1.9	3.68	<.0001
Emphy-Alg	1.07	1.96	1.2	.2006
LD-Alg	1.12	1.002	1.26	.0443
Gender (Male)	1.15	0.9	1.47	0.24
Age	1.6	1.3	1.97	<.0001
Pack years	1.06	1.03	1.1	0.0006

OR = odds ratio.

A multivariate logistic regression consisting of CCS-Alg, LD-Alg, Emphy-Alg was performed and resulted in an AUC of 0.648 (95% CI, 0.62–0.68) (S1 Fig in S1 Appendix).

The relationship between CVD mortality and age, gender and pack years was also assessed with a multivariate logistic regression, this model resulted in an AUC of 0.639 (95% CI, 0.61–0.67. AUC difference between the combined model and this one is 0.032 (95% CI, 0.03–0.033), AUC difference between the CCS-Alg, LD-Alg, Emphy-Alg model and this model is 0.009 (95% CI, 0.01–0.008)). this analysis demonstrated an increased CVD mortality risk of 1.94-fold (P < .0001) for every 10-year increase in age, and increased risk of 1.44-fold (P = 0.0019) for males and an increased risk of 1.073-fold (P = 0.0001) for every 10-year increase in pack years.

### CVD incidence

#### Univariate analyses

A statistically significant association between CCS-Alg categories and CVD incidence was found, with approximately 2.2-fold, and 5.9-fold increased risk of CVD incidence in categories 101–400, and >400 vs 0, respectively. When treating CCS-Alg as a continuous variable an increased 1.12-fold risk for CVD incidence was found for every 100 increment in the CCS-Alg score (Table 2). No statistically significant association was observed between Emphy-Alg score and CVD incidence (*P* = .176 S1 Table in S1 Appendix). However, a statistically significant inverse association was found with the LD-Alg score (1.1-fold difference [95% CI, 1.05–1.15] for each 10 HU decrements in the LD-Alg score; *P* < .0001 S1 Table in S1 Appendix).

#### Multivariate models

**Fig 2:**
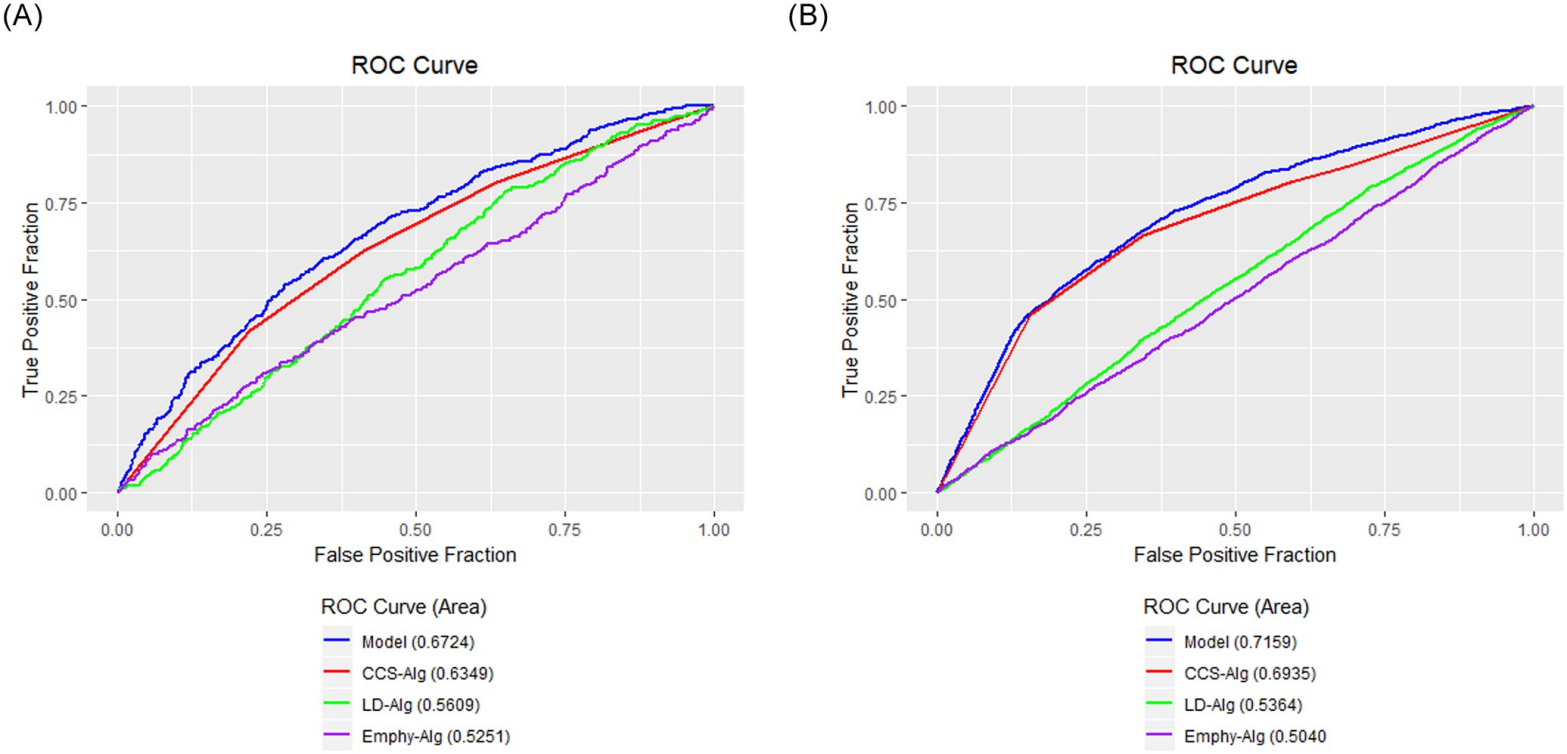
ROC curves with AUC for CCS-Alg, Emphy-Alg, LD-Alg univariate analyses and a multivariate model consisting of all the above together with age, gender and pack years depicting the discriminatory power of the scores for CVD mortality (A) and incidence (B). AUC difference comparing the Model AUC and CCS-Alg is 0.036 (95% CI, 0.037–0.035) for mortality (A) and 0.025 (95% CI, 0.024–0.026) for incidence (B).

The relationship between CVD incidence and CCS-Alg, Emphy-Alg, and LD-Alg was explored via a multivariate logistic regression (Table 4). The AUC for the overall model was 0.7159 (95% CI, 0.71–0.73), suggested moderate discrimination ability (Fig 2B). The analysis demonstrated an increased CVD incidence risk of approximately 1.96-fold, and 4.9-fold in CCS-Alg categories 101–400 and >400 vs 0. There was no increased risk in the 1–10 or 11–100 category vs 0. The analysis also demonstrated reduction of CVD incidence risk (0.89-fold difference) per 10% increments in Emphy-Alg score (Table 4).

**Table 4:** Multivariate analysis evaluating the association between CCS-Alg categories, Emphy-Alg, LD-Alg, age, gender, pack years and CVD incidence.

Level	OR estimate	95% confidence limits		*P*-value
CCS-Alg 1–10 vs 0	0.85	0.68	1.06	.149
CCS-Alg 11–100 vs 0	1.06	0.92	1.23	.426
CCS-Alg 101–400 vs 0	1.96	1.71	2.26	<.0001
CCS-Alg >400 vs 0	4.94	4.33	5.63	<.0001
Emphy-Alg	0.89	0.84	0.94	<.0001
LD-Alg	1.03	0.98	1.09	.181
Gender (Male)	1.32	1.2	1.46	<.0001
Age	1.24	1.14	1.36	<.0001
Pack years	1.05	1.03	1.07	<.0001

OR = odds ratio.

A multivariate logistic regression consisting of CCS-Alg, LD-Alg, Emphy-Alg was performed and resulted in an AUC of 0.699 (95% CI, 0.68–0.7) (S2 Fig in S1 Appendix).

The relationship between CVD incidence and age, gender and pack years was also assessed with a multivariate logistic regression, this model resulted in an AUC of 0.6288 (95% CI, 0.62–0.64, AUC difference between the combined model and this one is 0.08 (95% CI, 0.086–0.087), AUC difference between the CCS-Alg, LD-Alg, Emphy-Alg model and this model is 0.071 (95% CI, 0.07–0.071)). This analysis demonstrated an increased CVD incidence risk of 1.67-fold (P < .0001) for every 10-year increase in age, and increased risk of 1.81-fold (P < .0001) for males and an increased risk of 1.06-fold (P = 0.0001) for every 10-year increase in pack years.

## Discussion

The current analysis demonstrated that the automatic CT scan evaluation tools CCS-Alg, Emphy-Alg, and LD-Alg are statistically significant predictors for CVD mortality, with CCS-Alg providing the highest predictive value of these 3 tools. As for CVD incidence, our findings suggest that CCS-Alg and LD-Alg (but not Emphy-Alg) are statistically significant predictors.

Our findings show that CCS-Alg, Emphy-Alg, and LD-Alg are statistically significantly better predictors for CVD incidence than age, gender or pack year, which are by themselves well known predictors of both CVD mortality and or CVD incidence [20–23]. It can be seen that when adding all of the above predictors into one multivariate model we achieve an improved model with a statistically significant and higher AUC both for CVD mortality and for CVD incidence.

Our results with the CCS-Alg are similar in their prediction trend to results obtained using manual Agatston score categories. For example, Oudkerk et al. [24] have shown a rise in relative risk (RR) of 1.9 in the 1–100 category to 7.2 RR in 400–1000 while we show a rise in OR from 1.1 to 5.9 respectively. A study of the American College of Radiology Imaging Network (ACRIN) arm of the NLST which randomly selected 1575 cases and manually assessed CCS, showing that for Agatston scores of 1–100, 101–1000 and >1000 the hazard ratios were 1.27, 3.57 and 6.63, respectively. Our results show similar numbers while increasing the population and automating the process [25].

The advantage of CCS-Alg over manual processing of scans for quantifying calcifications in the coronary arteries is that this post-processing tool is fully automated utilizing machine learning and AI approaches. Multiple studies have shown the benefits of machine learning and AI to the medical field with respect to prediction of diagnosis [26], time saving [27] and better results in finding a certain pathology [28]. Furthermore, using automated tools such as CCS-Alg could help address a wider range of predictors of various diseases, even in cases where these diseases are not the focus/motivation of the screening. For example, in the case of NLST, patients were screened for lung cancer; however, the scans could also be used for identifying CVD risk. The automated process also enables the addition of these scores into a database in a reliable and efficient way. This will essentially help encode radiology reports into data that will allow a researcher or clinician to query the desired information that today is only available through manually looking at each report and hand picking the wanted data.

Our finding that LD-Alg and Emphy-Alg are statistically significant predictors of CVD mortality is consistent with prior studies showing that emphysema [29] and fatty liver [8–10] (on which Emphy-Alg and LD-Alg are based, respectively) are predictors of CVD mortality. However, surprisingly, for each 10% increment in the Emphy-Alg score, we observed reduced risk of CVD incidence.

All three of our algorithms are fully automated and provide additional and critical information to the physician. They all provide continuous exact measurements about the severity of the disease, as opposed to simply a category. This information can support the physician in better assessing CVD severity and consequently in better treatment decisions. For example, a CCS-Alg score of 401 is in the same Agatston category as a score of 802; however the probability of having a CVD incidence with a CCS of 802 is higher by approximately 25% during a median follow-up period of 3.8 years than that of a CCS of 401 [4].

Our study is limited by the lack of manual Agatston score on the NLST data. Therefore, the automatic tools could not be directly compared to the manual Agatston score procedure. However, we were able to show that the algorithms (particularly CCS-Alg) can predict CVD incidence and mortality even without a manual Agatston score. Our approach has a few limitations as well, by automating the process we increase the risk of overdiagnosis. While is still isn’t clear that the added information will influence management. However, it might also give the opportunity to approach a patient not by a single disease at a time but identify multiple risk factors with a single test.

In conclusion, our study demonstrated that automatic algorithms such as CCS-Alg, on its own or in combination with Emphy-Alg, LD-Alg, age, gender and pack years can be used in clinical practice as predictors of CVD incidence and mortality.

## Supporting information

S1 Appendix (DOCX)
