# Supplementary material for: Using machine learning algorithms to review computed tomography scans and assess risk for cardiovascular disease: Retrospective analysis from the National Lung Screening Trial (NLST)
Source: PLoS One. 2020 Aug 3;15(8):e0236021. doi: 10.1371/journal.pone.0236021 (PMC7398499; doi:10.1371/journal.pone.0236021)
Supplement: S1 Appendix — (DOCX) [file pone.0236021.s001.docx]

**S1 Fig –ROC curve with AUC for CCS-Alg, Emphy-Alg, LD-Alg multivariate model depicting the discriminatory power of the scores for CVD mortality. AUC = 0.648 (95% CI, 0.62-0.68).**


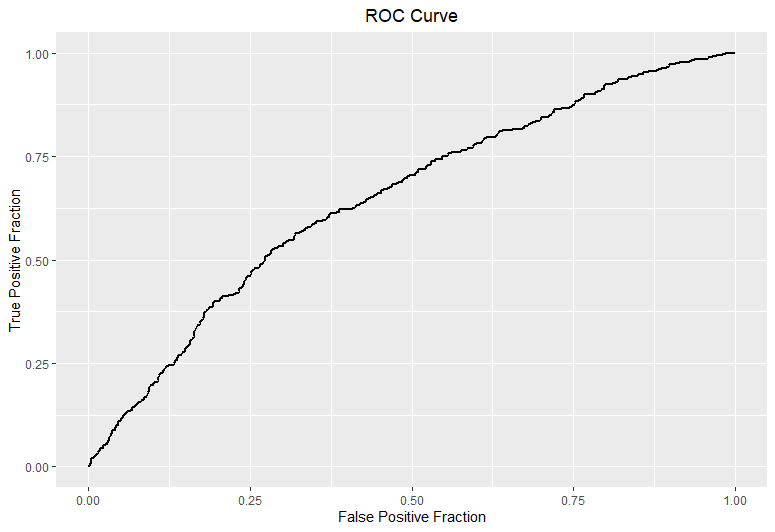


**S2 Fig - ROC curve with AUC for CCS-Alg, Emphy-Alg, LD-Alg multivariate model depicting the discriminatory power of the scores for CVD incidence. AUC = 0.699 (95% CI, 0.68-0.7).**
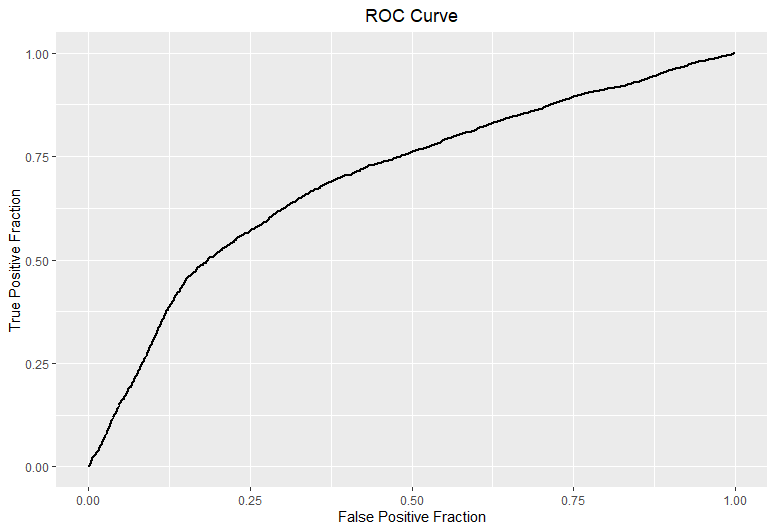


**S1 Table. Univariate analysis evaluating the association between Emphy-Alg, LD-Alg, age, gender and pack years with CVD mortality and incidence.**

|  | **Univariate variable** | **OR estimate** | **95% confidence limits** | | ***P-*value** |
| --- | --- | --- | --- | --- | --- |
| CVD mortality | Emphy-Alg | 1.14 | 1.03 | 1.26 | .0146 |
|  | LD-Alg | 1.16 | 1.04 | 1.29 | .0077 |
|  | Age | 1.07 | 1.05 | 1.09 | <.0001 |
|  | Gender - Male  Pack years | 1.59  1.01 | 1.27  1.00 | 2  1.01 | <.0001  <.0001 |
| CVD Incidence | Emphy-Alg | 0.96 | 0.91 | 1.02 | .176 |
|  | LD-Alg | 0.9 | 0.87 | 0.95 | <.0001 |
|  | Age | 1.06 | 1.05 | 1.07 | <.0001 |
|  | Gender - Male  Pack years | 1.93  1.009 | 1.78  1.008 | 2.12  1.01 | <.0001  <.0001 |

CVD = cardiovascular disease, OR = odds ratio

**Example of SAS code:**

We ran standard SAS procedures for all analyses. Mainly PROC LOGISTIC to calculate odds ratios, and generate AUC's with Wald confidence.

**proc** **logistic** data=cvd plots(only)=roc ;

class Death_CVD (ref=first) ccs1(ref=first) / param=ref ;

model Death_CVD = ccs1 emp_f mflk_f / cl clodds=wald ORPVALUE ;

units emp_f=**10** mflk_f=**10**;

roc 'CCS-Alg' ccs1 ;

roc 'Emphy-Alg' emp_f ;

roc 'LD-Alg' mflk_f ;

roccontrast / estimate e;

format ccs1 ccsf. ;

**run**;
